# The genome sequence of the Ingrailed Clay, *Diarsia mendica *(Fabricius, 1775)

**DOI:** 10.12688/wellcomeopenres.20106.1

**Published:** 2023-10-12

**Authors:** Douglas Boyes, Clare Boyes, University of Oxford and Wytham Woods Genome Acquisition Lab, Darwin Tree of Life Barcoding collective, Wellcome Sanger Institute Tree of Life programme, Wellcome Sanger Institute Scientific Operations: DNA Pipelines collective, Tree of Life Core Informatics collective, Darwin Tree of Life Consortium

**Affiliations:** 1 UK Centre for Ecology & Hydrology, Wallingford, England, UK; 2 Independent researcher, Welshpool, Wales, UK

**Keywords:** Diarsia mendica, Ingrailed Clay, genome sequence, chromosomal, Lepidoptera

## Abstract

We present a genome assembly from an individual male *Diarsia mendica* (the Ingrailed Clay; Arthropoda; Insecta; Lepidoptera; Noctuidae). The genome sequence is 727.9 megabases in span. Most of the assembly is scaffolded into 32 chromosomal pseudomolecules, including the Z sex chromosome. The mitochondrial genome has also been assembled and is 15.37 kilobases in length. Gene annotation of this assembly on Ensembl identified 14,077 protein coding genes.

## Species taxonomy

Eukaryota; Metazoa; Eumetazoa; Bilateria; Protostomia; Ecdysozoa; Panarthropoda; Arthropoda; Mandibulata; Pancrustacea; Hexapoda; Insecta; Dicondylia; Pterygota; Neoptera; Endopterygota; Amphiesmenoptera; Lepidoptera; Glossata; Neolepidoptera; Heteroneura; Ditrysia; Obtectomera; Noctuoidea; Noctuidae; Noctuinae; Noctuini; *Diarsia*; *Diarsia mendica* (Fabricius, 1775) (NCBI:txid987924).

## Background

*Diarsia mendica* (Ingrailed Clay) is a macro-moth in the family Noctuidae. The species has a southerly distribution in Britain and is mainly found throughout northern and central Europe; however there are a few records as far east as Siberia ( GBIF Secretariat, 2023). In the UK, the species has undergone a significant decline in abundance since the 1970s ( Randle *et al.*, 2019).

The moth is highly variable both within local populations, and across its range. Northern populations are generally darker and smaller, with recognised sub-species in Shetland and Orkney. *D. mendica* can usually be identified by the outline of the kidney mark and a black dot between the oval mark and the trailing edge of the forewing. The forewing length is 13–17 mm ( Waring *et al.*, 2017). The common name of this moth includes an heraldic term, ‘ingrailed’, indicating a decorative border ( Marren, 2019). Close examination reveals arrowhead-shaped marks along the outer edge of the forewing.

*D. mendica* has one generation a year, flying between late May and July in the southern part of its range and up to a month later in the north. It regularly comes to light; can be attracted by sugaring; and also feeds on flowers. The moth is found in a variety of habitats including woodland and gardens in the south of its range; but favouring heathlands and moorlands in the north. *D. mendica* overwinters as a small larva. It feeds at night on a range of herbaceous plants before pupating underground ( Waring *et al.*, 2017).

The genome sequence from *D. mendica* will be useful for research into colour variation in moths, and more generally for comparative studies across the Lepidoptera. The genome of *D. mendica* was sequenced as part of the Darwin Tree of Life Project, a collaborative effort to sequence all named eukaryotic species in the Atlantic Archipelago of Britain and Ireland. Here we present a chromosomally complete genome sequence for *D. mendica* based on one male specimen from Wytham Woods, Oxfordshire, UK.

## Genome sequence report

**Figure 1.**
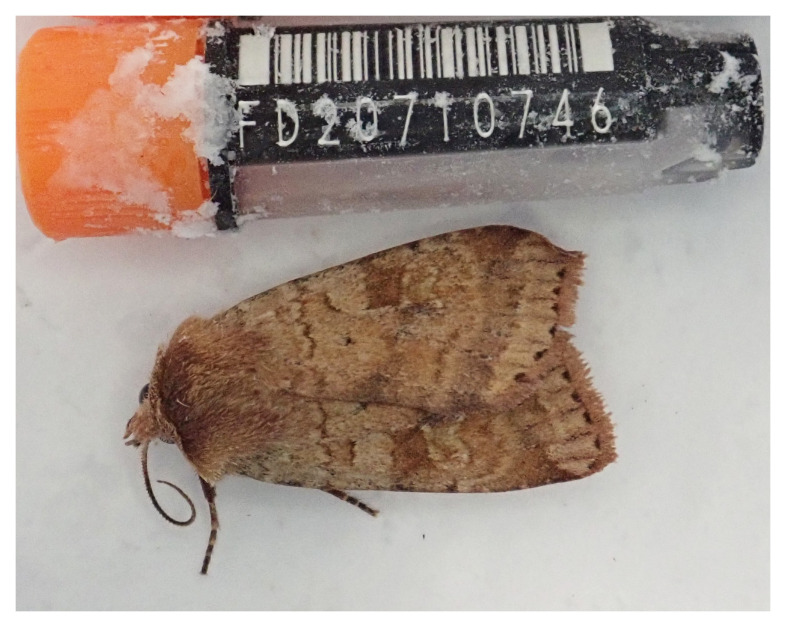
Photograph of the *Diarsia mendica* (ilDiaMeni1) specimen used for genome sequencing.

The genome was sequenced from one male *Diarsia mendica* ( Figure 1) collected from Wytham Woods, Oxfordshire, UK (51.77, –1.32). A total of 40-fold coverage in Pacific Biosciences single-molecule HiFi long reads was generated. Primary assembly contigs were scaffolded with chromosome conformation Hi-C data. Manual assembly curation corrected 58 missing joins or mis-joins and removed 33 haplotypic duplications, reducing the assembly length by 6.04% and the scaffold number by 33.96%, and decreasing the scaffold N50 by 3.29%.

**Figure 2.**
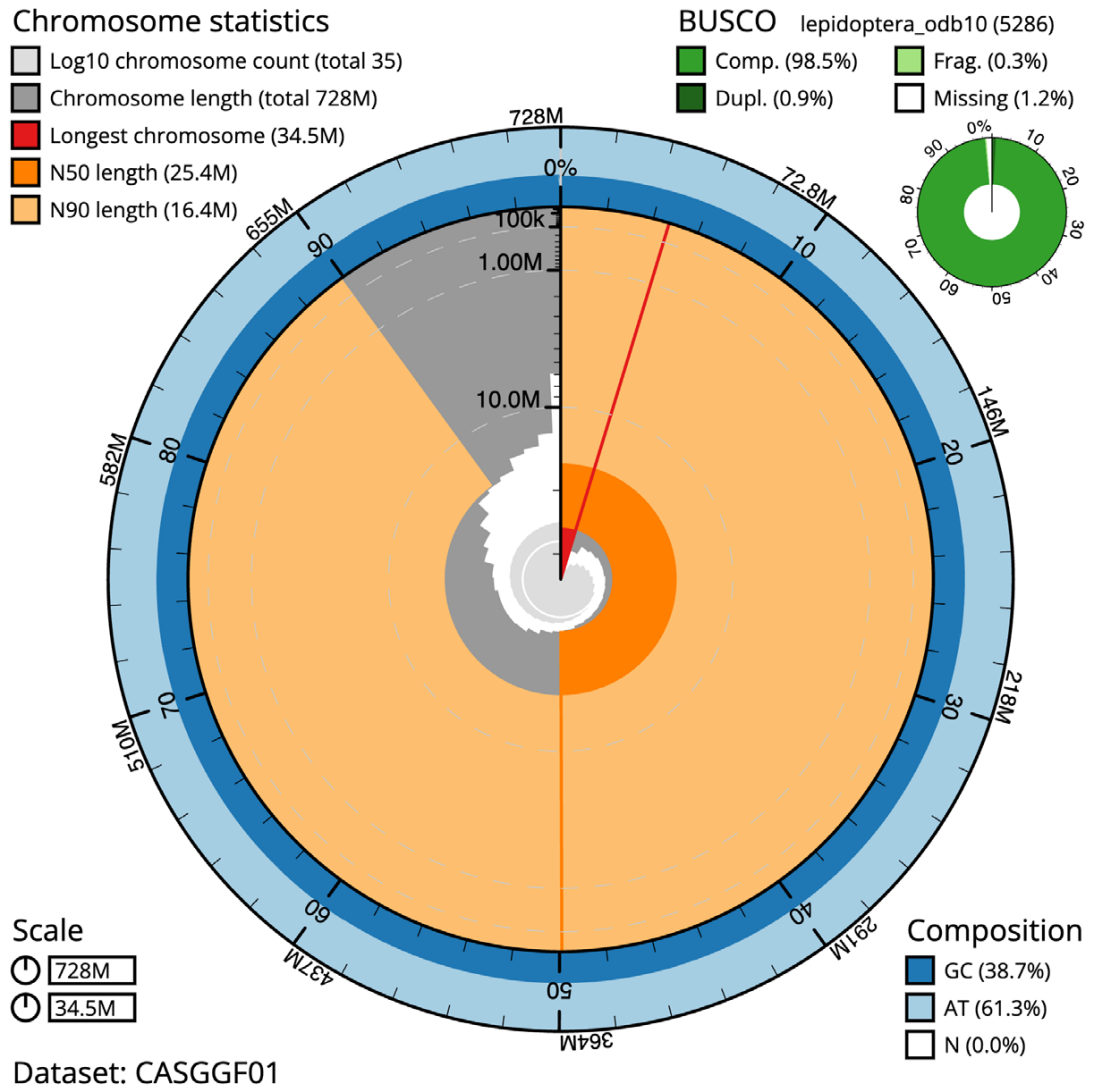
Genome assembly of *Diarsia mendica*, ilDiaMeni1.1: metrics. The BlobToolKit Snailplot shows N50 metrics and BUSCO gene completeness. The main plot is divided into 1,000 size-ordered bins around the circumference with each bin representing 0.1% of the 727,952,529 bp assembly. The distribution of scaffold lengths is shown in dark grey with the plot radius scaled to the longest scaffold present in the assembly (34,520,335 bp, shown in red). Orange and pale-orange arcs show the N50 and N90 scaffold lengths (25,354,251 and 16,374,863 bp), respectively. The pale grey spiral shows the cumulative scaffold count on a log scale with white scale lines showing successive orders of magnitude. The blue and pale-blue area around the outside of the plot shows the distribution of GC, AT and N percentages in the same bins as the inner plot. A summary of complete, fragmented, duplicated and missing BUSCO genes in the lepidoptera_odb10 set is shown in the top right. An interactive version of this figure is available at https://blobtoolkit.genomehubs.org/view/Diarsia%20mendica/dataset/CASGGF01/snail.

**Figure 3.**
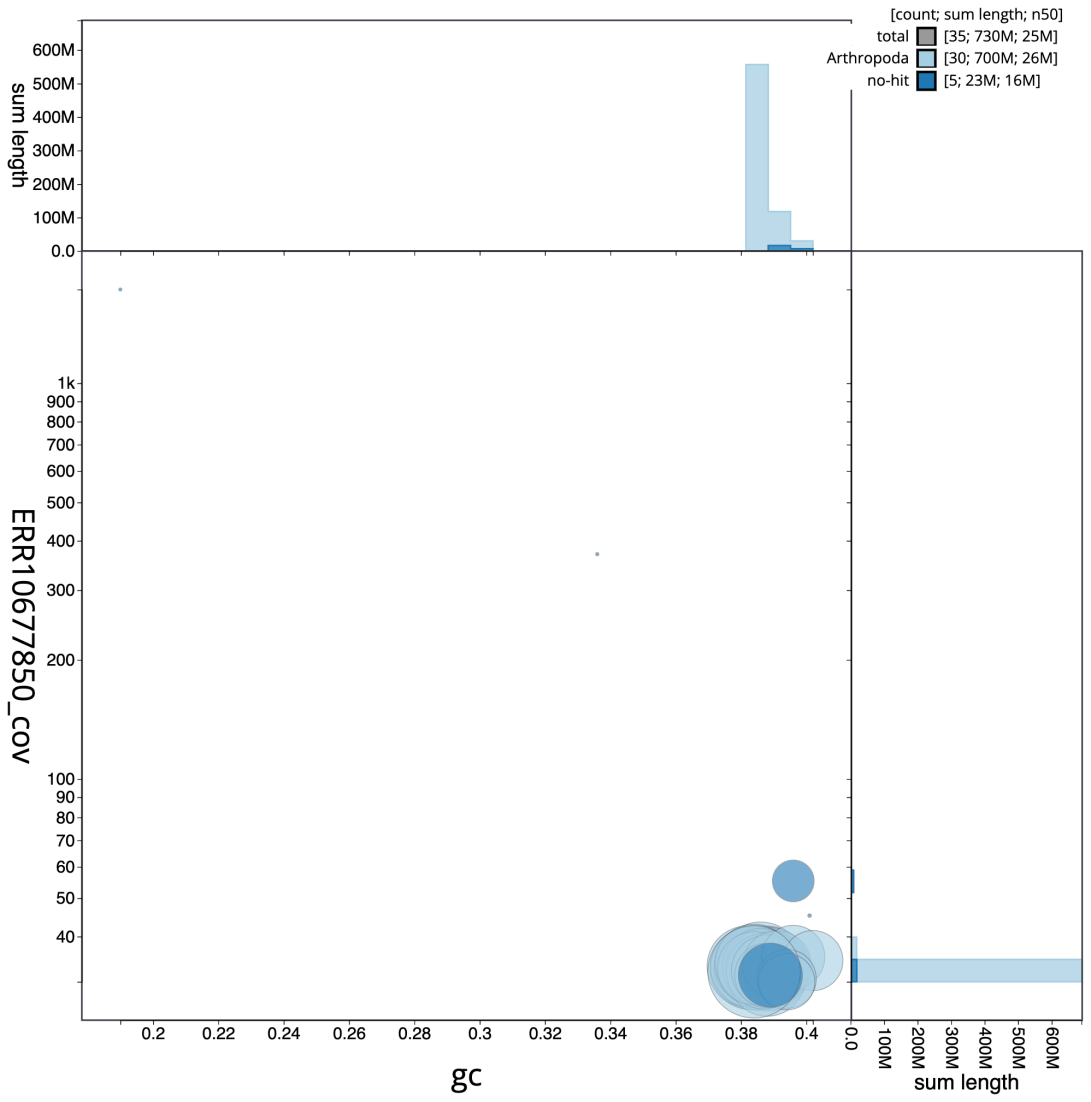
Genome assembly of *Diarsia mendica*, ilDiaMeni1.1: BlobToolKit GC-coverage plot. Scaffolds are coloured by phylum. Circles are sized in proportion to scaffold length. Histograms show the distribution of scaffold length sum along each axis. An interactive version of this figure is available at https://blobtoolkit.genomehubs.org/view/Diarsia%20mendica/dataset/CASGGF01/blob.

**Figure 4.**
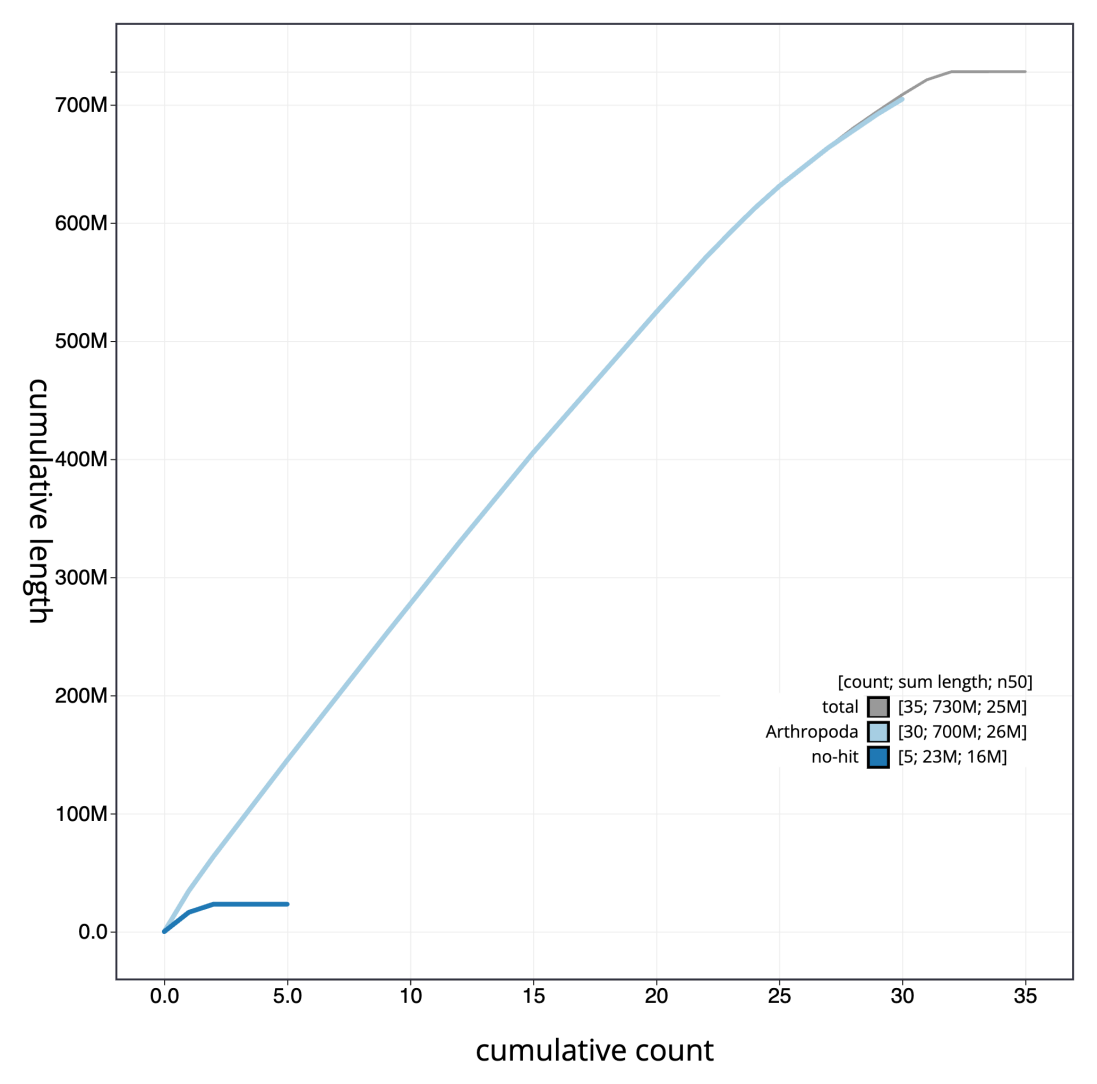
Genome assembly of *Diarsia mendica*, ilDiaMeni1.1: BlobToolKit cumulative sequence plot. The grey line shows cumulative length for all scaffolds. Coloured lines show cumulative lengths of scaffolds assigned to each phylum using the buscogenes taxrule. An interactive version of this figure is available at https://blobtoolkit.genomehubs.org/view/Diarsia%20mendica/dataset/CASGGF01/cumulative.

**Figure 5.**
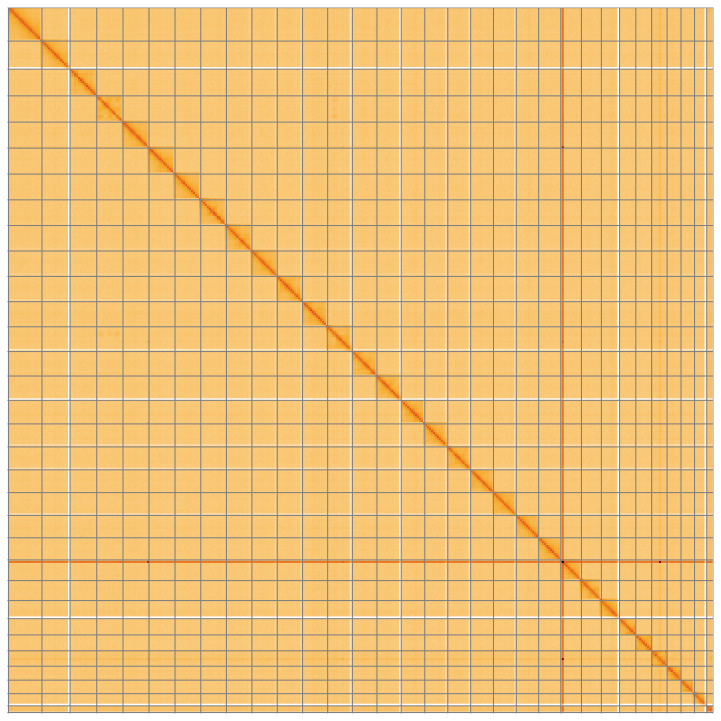
Genome assembly of *Diarsia mendica*, ilDiaMeni1.1: Hi-C contact map of the ilDiaMeni1.1 assembly, visualised using HiGlass. Chromosomes are shown in order of size from left to right and top to bottom. An interactive version of this figure may be viewed at https://genome-note-higlass.tol.sanger.ac.uk/l/?d=WNkhkJMKSum9Dm1JKAnxRg.

The final assembly has a total length of 727.9 Mb in 34 sequence scaffolds with a scaffold N50 of 25.4 Mb ( Table 1). A summary of the assembly statistics is shown in Figure 2, while the distribution of assembly scaffolds on GC proportion and coverage is shown in Figure 3. The cumulative assembly plot in Figure 4 shows curves for subsets of scaffolds assigned to different phyla. Most (99.99%) of the assembly sequence was assigned to 32 chromosomal-level scaffolds, representing 31 autosomes and the Z sex chromosome. The Z chromosome was identified based on synteny with *Diarsia rubi* (GCA_932274075.1) ( Boyes *et al.*, 2023). Chromosome 31/B1 is a putative supernumerary B chromosome. Alignments show lack of homology of B _1_ with assembled chromosomes of *Diarsia rubi*. Chromosome-scale scaffolds confirmed by the Hi-C data are named in order of size ( Figure 5; Table 2). While not fully phased, the assembly deposited is of one haplotype. Contigs corresponding to the second haplotype have also been deposited. The mitochondrial genome was also assembled and can be found as a contig within the multifasta file of the genome submission.

**Table 1.** Genome data for *Diarsia mendica*, ilDiaMeni1.1.

Project accession data		
Assembly identifier	ilDiaMeni1.1	
Assembly release date	2023-03-10	
Species	*Diarsia mendica*	
Specimen	ilDiaMeni1	
NCBI taxonomy ID	987924	
BioProject	PRJEB58240	
BioSample ID	SAMEA10979138	
Isolate information	ilDiaMeni1, male: thorax (DNA sequencing), head (Hi-C), abdomen (RNA sequencing)	
Assembly metrics *		*Benchmark*
Consensus quality (QV)	66.9	*≥ 50*
*k*-mer completeness	100%	*≥ 95%*
BUSCO **	C:98.9%[S:98.1%,D:0.8%],F:0.2%,M:0.9%,n:5,286	*C ≥ 95%*
Percentage of assembly mapped to chromosomes	99.99%	*≥ 95%*
Sex chromosomes	Z chromosome	*localised homologous pairs*
Organelles	Mitochondrial genome assembled	*complete single alleles*
Raw data accessions		
PacificBiosciences SEQUEL II	ERR10677850	
Hi-C Illumina	ERR10684077	
PolyA RNA-Seq Illumina	ERR11242513	
Genome assembly		
Assembly accession	GCA_949316265.1	
*Accession of alternate haplotype*	GCA_949316465.1	
Span (Mb)	727.9	
Number of contigs	152	
Contig N50 length (Mb)	7.6	
Number of scaffolds	34	
Scaffold N50 length (Mb)	25.4	
Longest scaffold (Mb)	34.5	
Genome annotation		
Number of protein-coding genes	14,077	
Number of non-coding genes	3,187	
Number of gene transcripts	26,300	

* Assembly metric benchmarks are adapted from column VGP-2020 of “Table 1: Proposed standards and metrics for defining genome assembly quality” from ( Rhie *et al.*, 2021). ** BUSCO scores based on the lepidoptera_odb10 BUSCO set using v5.3.2. C = complete [S = single copy, D = duplicated], F = fragmented, M = missing, n = number of orthologues in comparison. A full set of BUSCO scores is available at https://blobtoolkit.genomehubs.org/view/Diarsia%20mendica/dataset/CASGGF01/busco.

**Table 2.** Chromosomal pseudomolecules in the genome assembly of *Diarsia mendica*, ilDiaMeni1.

INSDC accession	Chromosome	Length (Mb)	GC%
OX438887.1	1	28.99	38.5
OX438888.1	2	27.56	38.5
OX438889.1	3	27.19	38.5
OX438890.1	4	26.87	38.5
OX438891.1	5	26.79	38.5
OX438892.1	6	26.58	39.0
OX438893.1	7	26.48	38.5
OX438894.1	8	26.34	38.0
OX438895.1	9	26.22	38.5
OX438896.1	10	26.08	38.5
OX438897.1	11	25.94	38.5
OX438898.1	12	25.55	39.0
OX438899.1	13	25.35	38.5
OX438900.1	14	25.06	38.5
OX438901.1	15	24.36	38.5
OX438902.1	16	23.74	39.0
OX438903.1	17	23.6	39.0
OX438904.1	18	23.57	38.5
OX438905.1	19	23.57	38.5
OX438906.1	20	23.1	39.0
OX438907.1	21	22.86	38.5
OX438908.1	22	21.38	38.5
OX438909.1	23	20.57	39.0
OX438910.1	24	18.73	39.0
OX438911.1	25	16.8	39.0
OX438912.1	26	16.37	39.0
OX438913.1	27	15.93	39.5
OX438914.1	28	14.43	40.0
OX438915.1	29	13.92	39.5
OX438916.1	30	12.61	39.5
OX438917.1	31	6.83	39.5
OX438886.1	Z	34.52	38.5
OX438918.1	MT	0.02	19.5

The estimated Quality Value (QV) of the final assembly is 66.9 with *k*-mer completeness of 100%, and the assembly has a BUSCO v5.3.2 completeness of 98.9% (single = 98.1%, duplicated = 0.8%), using the lepidoptera_odb10 reference set ( *n* = 5,286).

Metadata for specimens, spectral estimates, sequencing runs, contaminants and pre-curation assembly statistics can be found at https://links.tol.sanger.ac.uk/species/987924.

## Genome annotation report

The *Diarsia mendica* genome assembly (GCA_949316265.1) was annotated using the Ensembl rapid annotation pipeline ( Table 1; https://rapid.ensembl.org/Diarsia_mendica_GCA_949316265.1/Info/Index). The resulting annotation includes 26,300 transcribed mRNAs from 14,077 protein-coding and 3,187 non-coding genes.

## Methods

### Sample acquisition and nucleic acid extraction

A male *Diarsia mendica* (specimen ID Ox001878, individual ilDiaMeni1) was collected from Wytham Woods, Oxfordshire (biological vice-county Berkshire), UK (latitude 51.77, longitude –1.32) on 2021-05-28 using a light trap. The specimen was collected and identified by Douglas Boyes (University of Oxford) and preserved on dry ice.

The sample was prepared for DNA extraction at the Tree of Life laboratory, Wellcome Sanger Institute (WSI). The ilDiaMeni1 sample was weighed and dissected on dry ice with tissue set aside for Hi-C sequencing. Thorax tissue was cryogenically disrupted to a fine powder using a Covaris cryoPREP Automated Dry Pulveriser, receiving multiple impacts. DNA was extracted at the WSI Scientific Operations core using the Qiagen MagAttract HMW DNA kit, according to the manufacturer’s instructions.

RNA was extracted from abdomen tissue of ilDiaMeni1 in the Tree of Life Laboratory at the WSI using TRIzol, according to the manufacturer’s instructions. RNA was then eluted in 50 μl RNAse-free water and its concentration assessed using a Nanodrop spectrophotometer and Qubit Fluorometer using the Qubit RNA Broad-Range (BR) Assay kit. Analysis of the integrity of the RNA was done using Agilent RNA 6000 Pico Kit and Eukaryotic Total RNA assay.

### Sequencing

Pacific Biosciences HiFi circular consensus DNA sequencing libraries were constructed according to the manufacturers’ instructions. Poly(A) RNA-Seq libraries were constructed using the NEB Ultra II RNA Library Prep kit. DNA and RNA sequencing was performed by the Scientific Operations core at the WSI on Pacific Biosciences SEQUEL II (HiFi) and Illumina NovaSeq 6000 (RNA-Seq) instruments. Hi-C data were also generated from head tissue of ilDiaMeni1 using the Arima2 kit and sequenced on the Illumina NovaSeq 6000 instrument.

### Genome assembly, curation and evaluation

Assembly was carried out with Hifiasm ( Cheng *et al.*, 2021) and haplotypic duplication was identified and removed with purge_dups ( Guan *et al.*, 2020). The assembly was then scaffolded with Hi-C data ( Rao *et al.*, 2014) using YaHS ( Zhou *et al.*, 2023). The assembly was checked for contamination and as described previously ( Howe *et al.*, 2021). Manual curation was performed using HiGlass ( Kerpedjiev *et al.*, 2018) and Pretext ( Harry, 2022). The mitochondrial genome was assembled using MitoHiFi ( Uliano-Silva *et al.*, 2023), which runs MitoFinder ( Allio *et al.*, 2020) or MITOS ( Bernt *et al.*, 2013) and uses these annotations to select the final mitochondrial contig and to ensure the general quality of the sequence.

A Hi-C map for the final assembly was produced using bwa-mem2 ( Vasimuddin *et al.*, 2019) in the Cooler file format ( Abdennur & Mirny, 2020). To assess the assembly metrics, the *k*-mer completeness and QV consensus quality values were calculated in Merqury ( Rhie *et al.*, 2020). This work was done using Nextflow ( Di Tommaso *et al.*, 2017) DSL2 pipelines “sanger-tol/readmapping” ( Surana *et al.*, 2023a) and “sanger-tol/genomenote” ( Surana *et al.*, 2023b). The genome was analysed within the BlobToolKit environment ( Challis *et al.*, 2020) and BUSCO scores ( Manni *et al.*, 2021; Simão *et al.*, 2015) were calculated.

Table 3 contains a list of relevant software tool versions and sources.

**Table 3.** Software tools: versions and sources.

Software tool	Version	Source
BlobToolKit	4.1.5	https://github.com/blobtoolkit/blobtoolkit
BUSCO	5.3.2	https://gitlab.com/ezlab/busco
Hifiasm	0.16.1-r375	https://github.com/chhylp123/hifiasm
HiGlass	1.11.6	https://github.com/higlass/higlass
Merqury	MerquryFK	https://github.com/thegenemyers/MERQURY.FK
MitoHiFi	2	https://github.com/marcelauliano/MitoHiFi
PretextView	0.2	https://github.com/wtsi-hpag/PretextView
purge_dups	1.2.3	https://github.com/dfguan/purge_dups
sanger-tol/genomenote	v1.0	https://github.com/sanger-tol/genomenote
sanger-tol/readmapping	1.1.0	https://github.com/sanger-tol/readmapping/tree/1.1.0
YaHS	1.2a	https://github.com/c-zhou/yahs

### Genome annotation

The Ensembl gene annotation system ( Aken *et al.*, 2016) was used to generate annotation for the *Diarsia mendica* assembly (GCA_949316265.1). Annotation was created primarily through alignment of transcriptomic data to the genome, with gap filling via protein-to-genome alignments of a select set of proteins from UniProt ( UniProt Consortium, 2019).

### Wellcome Sanger Institute – Legal and Governance

The materials that have contributed to this genome note have been supplied by a Darwin Tree of Life Partner. The submission of materials by a Darwin Tree of Life Partner is subject to the **‘Darwin Tree of Life Project Sampling Code of Practice’**, which can be found in full on the Darwin Tree of Life website here. By agreeing with and signing up to the Sampling Code of Practice, the Darwin Tree of Life Partner agrees they will meet the legal and ethical requirements and standards set out within this document in respect of all samples acquired for, and supplied to, the Darwin Tree of Life Project. 

Further, the Wellcome Sanger Institute employs a process whereby due diligence is carried out proportionate to the nature of the materials themselves, and the circumstances under which they have been/are to be collected and provided for use. The purpose of this is to address and mitigate any potential legal and/or ethical implications of receipt and use of the materials as part of the research project, and to ensure that in doing so we align with best practice wherever possible. The overarching areas of consideration are:

• Ethical review of provenance and sourcing of the material

• Legality of collection, transfer and use (national and international) 

Each transfer of samples is further undertaken according to a Research Collaboration Agreement or Material Transfer Agreement entered into by the Darwin Tree of Life Partner, Genome Research Limited (operating as the Wellcome Sanger Institute), and in some circumstances other Darwin Tree of Life collaborators.

## Data Availability

European Nucleotide Archive: *Diarsia mendica* (ingrailed clay). Accession number PRJEB58240; https://identifiers.org/ena.embl/PRJEB58240. ( Wellcome Sanger Institute, 2023) The genome sequence is released openly for reuse. The *Diarsia mendica* genome sequencing initiative is part of the Darwin Tree of Life (DToL) project. All raw sequence data and the assembly have been deposited in INSDC databases. Raw data and assembly accession identifiers are reported in Table 1.

## Author information

Members of the University of Oxford and Wytham Woods Genome Acquisition Lab are listed here: https://doi.org/10.5281/zenodo.4789928.

Members of the Darwin Tree of Life Barcoding collective are listed here: https://doi.org/10.5281/zenodo.4893703.

Members of the Wellcome Sanger Institute Tree of Life programme are listed here: https://doi.org/10.5281/zenodo.4783585.

Members of Wellcome Sanger Institute Scientific Operations: DNA Pipelines collective are listed here: https://doi.org/10.5281/zenodo.4790455.

Members of the Tree of Life Core Informatics collective are listed here: https://doi.org/10.5281/zenodo.5013541.

Members of the Darwin Tree of Life Consortium are listed here: https://doi.org/10.5281/zenodo.4783558.

## References

[ref-1] Abdennur N Mirny LA : Cooler: Scalable storage for Hi-C data and other genomically labeled arrays. *Bioinformatics.* 2020;36(1):311–316. 10.1093/bioinformatics/btz540 31290943 PMC8205516

[ref-2] Aken BL Ayling S Barrell D : The Ensembl gene annotation system. *Database (Oxford).* 2016;2016: baw093. 10.1093/database/baw093 27337980 PMC4919035

[ref-3] Allio R Schomaker‐Bastos A Romiguier J : MitoFinder: Efficient automated large‐scale extraction of mitogenomic data in target enrichment phylogenomics. *Mol Ecol Resour.* 2020;20(4):892–905. 10.1111/1755-0998.13160 32243090 PMC7497042

[ref-4] Bernt M Donath A Jühling F : MITOS: Improved *de novo* metazoan mitochondrial genome annotation. *Mol Phylogenet Evol.* 2013;69(2):313–319. 10.1016/j.ympev.2012.08.023 22982435

[ref-5] Boyes D Holland PWH, University of Oxford and Wytham Woods Genome Acquisition Lab : The genome sequence of the Small Square-spot, *Diarsia rubi* (Vieweg, 1790) [version 1; peer review: awaiting peer review]. *Wellcome Open Res.* 2023;8:210. 10.12688/wellcomeopenres.19299.1 PMC11358679 39211527

[ref-6] Challis R Richards E Rajan J : BlobToolKit - interactive quality assessment of genome assemblies. *G3 (Bethesda).* 2020;10(4):1361–1374. 10.1534/g3.119.400908 32071071 PMC7144090

[ref-7] Cheng H Concepcion GT Feng X : Haplotype-resolved *de novo* assembly using phased assembly graphs with hifiasm. *Nat Methods.* 2021;18(2):170–175. 10.1038/s41592-020-01056-5 33526886 PMC7961889

[ref-8] Di Tommaso P Chatzou M Floden EW : Nextflow enables reproducible computational workflows. *Nat Biotechnol.* 2017;35(4):316–319. 10.1038/nbt.3820 28398311

[ref-9] GBIF Secretariat: *Diarsia mendica* Fabricius, 1775. *GBIF Backbone Taxonomy.* 2023; [Accessed 25 June 2023]. Reference Source

[ref-10] Guan D McCarthy SA Wood J : Identifying and removing haplotypic duplication in primary genome assemblies. *Bioinformatics.* 2020;36(9):2896–2898. 10.1093/bioinformatics/btaa025 31971576 PMC7203741

[ref-11] Harry E : PretextView (Paired REad TEXTure Viewer): A desktop application for viewing pretext contact maps. 2022; [Accessed 19 October 2022]. Reference Source

[ref-12] Howe K Chow W Collins J : Significantly improving the quality of genome assemblies through curation. *GigaScience.* Oxford University Press,2021;10(1): giaa153. 10.1093/gigascience/giaa153 33420778 PMC7794651

[ref-13] Kerpedjiev P Abdennur N Lekschas F : HiGlass: web-based visual exploration and analysis of genome interaction maps. *Genome Biol.* 2018;19(1): 125. 10.1186/s13059-018-1486-1 30143029 PMC6109259

[ref-14] Manni M Berkeley MR Seppey M : BUSCO update: Novel and streamlined workflows along with broader and deeper phylogenetic coverage for scoring of eukaryotic, prokaryotic, and viral genomes. *Mol Biol Evol.* 2021;38(10):4647–4654. 10.1093/molbev/msab199 34320186 PMC8476166

[ref-15] Marren P : Emperors, Admirals, and Chimney Sweepers.Toller Fratrum, Dorset: Little Toller,2019. Reference Source

[ref-16] Randle Z Evans-Hill LJ Parsons MS : Atlas of Britain & Ireland’s Larger Moths.Newbury: NatureBureau,2019. Reference Source

[ref-17] Rao SSP Huntley MH Durand NC : A 3D map of the human genome at kilobase resolution reveals principles of chromatin looping. *Cell.* 2014;159(7):1665–1680. 10.1016/j.cell.2014.11.021 25497547 PMC5635824

[ref-18] Rhie A McCarthy SA Fedrigo O : Towards complete and error-free genome assemblies of all vertebrate species. *Nature.* 2021;592(7856):737–746. 10.1038/s41586-021-03451-0 33911273 PMC8081667

[ref-19] Rhie A Walenz BP Koren S : Merqury: Reference-free quality, completeness, and phasing assessment for genome assemblies. *Genome Biol.* 2020;21(1): 245. 10.1186/s13059-020-02134-9 32928274 PMC7488777

[ref-20] Simão FA Waterhouse RM Ioannidis P : BUSCO: assessing genome assembly and annotation completeness with single-copy orthologs. *Bioinformatics.* 2015;31(19):3210–3212. 10.1093/bioinformatics/btv351 26059717

[ref-21] Surana P Muffato M Qi G : sanger-tol/readmapping: sanger-tol/readmapping v1.1.0 - Hebridean Black (1.1.0). *Zenodo.* 2023a; [Accessed 21 July 2023]. 10.5281/zenodo.7755665

[ref-22] Surana P Muffato M Sadasivan Baby C : sanger-tol/genomenote (v1.0.dev). *Zenodo.* 2023b; [Accessed 21 July 2023]. Reference Source

[ref-23] Uliano-Silva M Ferreira JGRN Krasheninnikova K : MitoHiFi: a python pipeline for mitochondrial genome assembly from PacBio high fidelity reads. *BMC Bioinformatics.* 2023;24(1): 288. 10.1186/s12859-023-05385-y 37464285 PMC10354987

[ref-24] UniProt Consortium: UniProt: a worldwide hub of protein knowledge. *Nucleic Acids Res.* 2019;47(D1):D506–D515. 10.1093/nar/gky1049 30395287 PMC6323992

[ref-25] Vasimuddin M Misra S Li H : Efficient Architecture-Aware Acceleration of BWA-MEM for Multicore Systems.In: *2019 IEEE International Parallel and Distributed Processing Symposium (IPDPS).*IEEE,2019;314–324. 10.1109/IPDPS.2019.00041

[ref-26] Waring P Townsend M Lewington R : Field Guide to the Moths of Great Britain and Ireland: Third Edition.Bloomsbury Wildlife Guides,2017. Reference Source

[ref-28] Wellcome Sanger Institute: The genome sequence of the Ingrailed Clay, *Diarsia mendica* (Fabricius, 1775). European Nucleotide Archive.[dataset], accession number PRJEB58240,2023. 10.12688/wellcomeopenres.20106.1 PMC10850848 38333733

[ref-27] Zhou C McCarthy SA Durbin R : YaHS: yet another Hi-C scaffolding tool. *Bioinformatics.* 2023;39(1):btac808. 10.1093/bioinformatics/btac808 36525368 PMC9848053

