# Supplementary material for: The genome sequence of the Ingrailed Clay, Diarsia mendica (Fabricius, 1775)
Source: Wellcome Open Res. 2023 Oct 12;8:448. [Version 1] doi: 10.12688/wellcomeopenres.20106.1 (PMC10850848; doi:10.12688/wellcomeopenres.20106.1)
Supplement: Peer review history

# Reviewer report: Reviewer response for version 1

**DOI:** 10.21956/wellcomeopenres.22265.r72170

**Published:** 2024-02-07

**Authors:** Xiangyu Hao

**Affiliations:** 1 College of Life Sciences, Northwest A&F University, Yangling, Shaanxi, China

The manuscript provides a thorough and well-documented genome assembly and annotation of *Diarsia mendica*. It provides high-quality genome resources for Darwin Tree of Life Project. I suggest that the abstract could be improved by adding a brief sentence on the potential applications or significance of the genome assembly for future research.

Are sufficient details of methods and materials provided to allow replication by others?

Yes

Is the rationale for creating the dataset(s) clearly described?

Yes

Are the datasets clearly presented in a useable and accessible format?

Yes

Are the protocols appropriate and is the work technically sound?

Yes

Reviewer Expertise:

Zoology, Entomology, Genomics

I confirm that I have read this submission and believe that I have an appropriate level of expertise to confirm that it is of an acceptable scientific standard.

# Reviewer report: Reviewer response for version 1

**DOI:** 10.21956/wellcomeopenres.22265.r72161

**Published:** 2024-01-11

**Authors:** Kay Lucek

**Affiliations:** 1 Department for Environmental Sciences, University of Basel, Basel, Switzerland

This is the data note for the genome assembly of Diarsia mendica, which provides a great resource for future comparative studies, especially on the evolution of B chromosomes if this holds up.

 Overall the genome sequencing and assembly follow the standard approaches of the Darwin Tree of Life project. However, for a full replication of the assembly methods, one would need the specific settings of all the programs that were used for assembly and annotation.

 Concerning the annotation, it would be great if the authors could elaborate what fraction of the genes of the genome were actually annotated. Interestingly, they used an abdomen for RNAseq, which also contains the gut and therefore many exogenous transcripts. Similarly, transcripts in other body parts such as the brain, may have been missing.

Are sufficient details of methods and materials provided to allow replication by others?

Partly

Is the rationale for creating the dataset(s) clearly described?

Yes

Are the datasets clearly presented in a useable and accessible format?

Yes

Are the protocols appropriate and is the work technically sound?

Yes

Reviewer Expertise:

Speciation, Genomics, Evolutionary Biology, Lepidoptera

I confirm that I have read this submission and believe that I have an appropriate level of expertise to confirm that it is of an acceptable scientific standard.
